# Two or more dexamethasone intravitreal implants as monotherapy or in combination therapy for macular edema in retinal vein occlusion: subgroup analysis of a retrospective chart review study

**DOI:** 10.1186/s12886-015-0018-y

**Published:** 2015-04-01

**Authors:** Michael A Singer, Antonio Capone Jr, Pravin U Dugel, Richard F Dreyer, David G Dodwell, Daniel B Roth, Rui Shi, John G Walt, Lanita C Scott, David A Hollander, for the SHASTA Study Group

**Affiliations:** Medical Center Ophthalmology Associates, 9157 Huebner Road, San Antonio, TX 78240 USA; Associated Retinal Consultants, Novi, MI USA; Retinal Consultants of Arizona, Phoenix, AZ USA; Retina Northwest PC, Portland, OR USA; Illinois Retina Center, Springfield, IL USA; Retina-Vitreous Center, Rutgers Robert Wood Johnson Medical School, New Brunswick, NJ USA; Allergan, Inc., Irvine, CA USA

**Keywords:** Anti-VEGF, Corticosteroid, Dexamethasone, Implant, Intraocular pressure, Retinal vein occlusion, Steroid

## Abstract

**Background:**

Dexamethasone intravitreal implant (DEX implant) is a sustained-release biodegradable implant approved for treatment of macular edema associated with retinal vein occlusion (RVO). The safety and efficacy of treatment of RVO-associated macular edema with sequential DEX implants in clinical practice was evaluated in patients who received DEX implant as monotherapy compared with patients who received DEX implant in combination with other RVO treatments.

**Methods:**

A multicenter, retrospective, open-label chart review study (one study eye/patient) evaluated use of DEX implant and outcomes in 289 patients with branch or central RVO who received at least 2 DEX implant treatments in the study eye. Data were collected from the time of the first implant (baseline) to 3–6 months after the last implant. Subgroup analysis evaluated outcomes in patients receiving only DEX implant during the study versus patients receiving DEX implant plus adjunctive RVO treatments. Endpoints included best-corrected visual acuity (BCVA) and central retinal thickness (CRT) change from baseline.

**Results:**

DEX implant was used as monotherapy in 84 (29.1%) patients and in combination with other therapy in 205 (70.9%) patients. Mean number of DEX implant treatments received was 3.1 in the monotherapy group and 3.3 in the combination therapy group (*P* = 0.344). Mean time between implants was longer in the combination therapy group (177 vs. 151 days, *P* < 0.001). Mean change from baseline BCVA after the first through sixth DEX implants ranged from +0.6 to +3.4 lines in the monotherapy group and +1.3 to +2.8 lines in the combination therapy group. Mean decrease from baseline CRT ranged from 165 to 230 μm in the monotherapy group and 136 to 175 μm in the combination therapy group. Increased intraocular pressure was more common in the combination therapy group.

**Conclusions:**

Treatment of RVO-associated macular edema with at least 2 sequential DEX implants was safe and effective both when used alone and when combined with other RVO treatments. Improvements in BCVA and CRT were generally similar in the monotherapy and combined therapy groups.

**Trial registration:**

ClinicalTrials.gov NCT01411696.

## Background

Macular edema (ME) is a frequent cause of vision loss following retinal vein occlusion (RVO) [1,2]. Treatment options for ME after branch retinal vein occlusion (BRVO) include laser photocoagulation [3], intravitreal corticosteroids [4,5], and intravitreal vascular endothelial growth factor antagonists (anti-VEGF) [6]. Intravitreal corticosteroids [5,7] and anti-VEGF [8,9] are also effective in the treatment of ME associated with central retinal vein occlusion (CRVO). Early intervention and initiation of treatment for ME related to RVO is associated with better visual outcomes [10-12].

Dexamethasone intravitreal implant (DEX implant; Ozurdex; Allergan, Inc., Irvine, CA) is a sustained-release biodegradable implant used in the treatment of RVO-associated ME. DEX implant provides slow release of dexamethasone into the vitreous over a period of up to 6 months [13]. Phase 3 trials demonstrated the efficacy and safety of a single DEX implant and retreatment after 6 months in patients with BRVO and CRVO [5,14]. The optimal retreatment interval for DEX implant has not yet been determined.

We recently investigated patterns of DEX implant use in clinical practice in a retrospective chart review study of 289 patients who were treated with 2 or more implants for RVO-associated ME [15]. Repeat treatment with DEX implant resulted in significant improvement in best-corrected visual acuity (BCVA) and central retinal thickness (CRT) by optical coherence tomography (OCT) after each subsequent DEX implant injection, from the first to the sixth implant (*P* ≤ 0.037). Overall, 62.9% of patients gained at least 2 lines of BCVA during the study, and 48.1% gained at least 3 lines. Only 1.7% of patients required incisional glaucoma surgery.

Most of the patients in the study had been treated for complications of RVO prior to their first DEX implant [15]. Further, although some patients received only DEX implant during the study period, most received additional RVO treatments, most commonly intravitreal anti-VEGF therapy. Therefore, the efficacy and safety results reported could have been confounded by the effects of other RVO treatments. The purpose of the present analysis was to determine the efficacy, safety, and reinjection interval of DEX implant in the subgroup of patients who received DEX implant alone, as well as in the subgroup of patients who received DEX implant in combination with other RVO treatments.

## Methods

This is a subgroup analysis of results from SHASTA, a multicenter, retrospective, open-label chart review study. The study adhered to the tenets of the Declaration of Helsinki, was compliant with the Health Insurance Portability and Accountability Act, and was approved at each site by the New England Institutional Review Board, the UC Davis Institutional Review Board, or the Wake Forest University Health Sciences Institutional Review Board. All patients provided informed consent. The study is registered with the identifier NCT01411696 at ClinicalTrials.gov.

The study protocol was described in detail previously [15] and is summarized here. Patients at least 18 years of age diagnosed with ME secondary to RVO in the study eye, who had received at least 2 injections of DEX implant 0.7 mg in the study eye, and who had follow-up data available for at least 3 months after the last DEX implant were selected for the study. Patients who had received DEX implant previously as part of or during a clinical study were excluded. Concomitant adjunctive treatments and procedures for RVO were allowed. If both eyes met study eligibility criteria, the eye that had received the largest number of DEX implants was selected as the study eye.

Data collected from patient records from the time of the first implant (baseline) through follow up of at least 3 months and up to 6 months after the last implant included BCVA, CRT by OCT, DEX implant injections, other treatments and procedures for ME, intraocular pressure (IOP), cataract and glaucoma surgeries, and adverse events. Demographic and ophthalmic history data were collected from records of the baseline visit.

Data analysis followed the statistical plan previously described for results in the total study population [15]. Snellen BCVA measurements were converted to approximate Early Treatment Diabetic Retinopathy Study (ETDRS) letter scores for analysis, with 5 ETDRS letters = 1 line in BCVA. When measurements of BCVA or CRT were recorded at multiple visits between DEX implant injections, the measurement of peak treatment effect (best BCVA or thinnest CRT) was used in the analysis. Subgroup analysis evaluated results in patients who received DEX implant and no other RVO treatments (monotherapy group) and patients who received DEX implant in combination with other RVO treatments (combination therapy group) during the study period from the initial DEX implant to up to 6 months after the last DEX implant, similarly to the previously reported subgroup analysis of results by patient diagnosis (BRVO or CRVO) [15]. Results after each DEX implant injection are reported for the first through sixth implants, as only 10 patients received 7 or more DEX implant treatments. Observed values were used in all analyses.

Statistical analyses were performed with SAS version 9.2 (SAS Institute Inc., Cary, NC, USA) and a 2-sided alpha level of 0.05. Comparisons of the proportion of patients with an increase of at least 2 or 3 lines in BCVA from baseline used Cochran-Mantel-Haenszel tests adjusting for patient diagnosis (BRVO or CRVO). Analysis of other categorical variables used Pearson chi-square tests or Fisher exact tests. Analysis of covariance was used for the analysis of changes in BCVA and CRT from baseline with fixed effects of subgroup and diagnosis (BRVO or CRVO) and baseline BCVA or CRT as the covariate in each model. Comparisons of other continuous variables used *t*-tests. The Pearson correlation coefficient was used to evaluate the relationship between number of intravitreal injections and increased IOP.

## Results

Of the 289 patients in the study, 84 (29.1%) received DEX implant monotherapy during the study period and 205 (70.9%) received DEX implant in conjunction with other adjunctive treatment for ME associated with RVO. Baseline characteristics of the patients in the monotherapy and combination therapy groups are listed in Table 1. RVO diagnosis was similar in the groups. In the monotherapy group, 52.4% of patients had BRVO and 47.6% had CRVO, and in the combination therapy group, 55.1% of patients had BRVO and 44.9% had CRVO.

**Table 1:** **Baseline characteristics of patients and study eyes**

**Characteristic**	**Monotherapy n = 84**	**Combination therapy n = 205**	***P*** **value**
Mean (SD) age, years	72.6 (10.3)	71.7 (11.2)	0.515
Range	39–91	39–94	
Sex, n (%)			0.266
Female	44 (52.4)	122 (59.5)	
Male	40 (47.6)	83 (40.5)	
Race/Ethnicity, n (%)			0.007
White	34 (40.5)	108 (52.7)	
Black	2 (2.4)	7 (3.4)	
Asian	6 (7.1)	1 (0.5)	
Other	0 (0.0)	1 (0.5)	
Not recorded in chart	42 (50.0)	88 (42.9)	
Diagnosis, n (%)			0.671
BRVO	44 (52.4)	113 (55.1)	
CRVO	40 (47.6)	92 (44.9)	
Mean (SD) duration of ME, months	25.6 (26.4)	15.5 (21.4)	<0.001
Range	0–100	0–150	
Previous treatment for RVO, n (%)	70 (83.3)	178 (86.8)	0.439
Intravitreal anti-VEGF, n (%)	51 (60.7)	154 (75.1)	0.014
Bevacizumab	44 (52.4)	137 (66.8)	
Ranibizumab	11 (13.1)	29 (14.1)	
Pegaptanib	0 (.0)	3 (1.5)	
Ranibizumab and/or bevacizumab	51 (60.7)	152 (74.1)	
Mean (SD) number of ranibizumab and/or bevacizumab injections in these patients	5.2 (4.6)	4.5 (5.1)	0.383
Intravitreal triamcinolone, n (%)	44 (52.4)	71 (34.6)	0.005
Laser photocoagulation, n (%)	43 (51.2)	69 (33.7)	0.005
Focal	33 (39.3)	52 (25.4)	0.018
Panretinal	15 (17.9)	30 (14.6)	0.493
Pars plana vitrectomy, n (%)	12 (14.3)	25 (12.2)	0.629
Glaucoma or OHT at baseline, n (%)			0.438
Yes	27 (32.1)	64 (31.2)	
No	40 (47.6)	111 (54.1)	
Not recorded in chart	17 (20.2)	30 (14.6)	
Using IOP-lowering medication at baseline, n (%)	20 (23.8)	54 (26.3)	0.505
History of IOP response to steroid, n (%)			0.065
Yes	12 (14.3)	33 (16.1)	
No	42 (50.0)	126 (61.5)	
Not recorded in chart	30 (35.7)	46 (22.4)	
Lens status, n (%)			0.069
Phakic	29 (34.5)	99 (48.3)	
Pseudophakic	54 (64.3)	104 (50.7)	
Not recorded in chart	1 (1.2)	2 (1.0)	
Mean (SD) BCVA, lines	9.1 (4.7)	10.1 (4.6)	0.095
Snellen	20/125	20/100	
Mean (SD) CRT, μm	465 (177)	427 (183)	0.136

Abbreviations: *SD* standard deviation, *BRVO* branch retinal vein occlusion, *CRVO* central retinal vein occlusion, *ME* macular edema, *VEGF* vascular endothelial growth factor, *OHT* ocular hypertension, *IOP* intraocular pressure, *BCVA* best-corrected visual acuity, *CRT* central retinal thickness.

The mean duration of ME prior to the first DEX implant was significantly longer in the monotherapy group than in the combination therapy group (25.6 vs. 15.5 months, *P* < 0.001). Although a similar percentage of patients in each group (monotherapy, 83.3%; combination therapy, 86.8%) had received previous treatment for complications of RVO before their first DEX implant, patients in the monotherapy group were more likely to have received previous laser therapy, whereas patients in the combination therapy group were more likely to have received previous anti-VEGF therapy (Table 1).

The mean number of anti-VEGF injections before the first DEX implant was similar in the monotherapy group (3.2) and the combination therapy group (3.4). After starting DEX implant treatment, the mean number of anti-VEGF injections received decreased to 0 (as per definition) in the monotherapy group and to 2.7 in the combination therapy group.

### Treatment

The mean (standard deviation, SD) number of DEX implant treatments received by patients was 3.1 (1.5) in the monotherapy group and 3.3 (1.4) in the combination therapy group and did not differ significantly between groups (*P* = 0.344). The mean interval between DEX implant injections, calculated per patient, was slightly shorter in the monotherapy group than in the combination therapy group (151 vs. 177 days), and this difference was statistically significant (*P* < 0.001).

During the study period, 90.7% of patients in the combination therapy group received intravitreal anti-VEGF (mean of 3.0 bevacizumab or ranibizumab injections) (Table 2). The mean time to the first anti-VEGF injection in these patients was 180 days (median, 136 days) [15]. Laser treatment was received during the study period by 35.1% of patients in the combination therapy group, and 9 (4.4%) were treated with intravitreal triamcinolone (mean of 1.6 injections) during the study period (Table 2).

**Table 2:** **Treatments for complications of retinal vein occlusion administered during the study period**

**Treatment, n (%)**	**Monotherapy n = 84**	**Combination therapy n = 205**
DEX implant	84 (100)	205 (100)
Any other treatment	0 (0)	205 (100)
Intravitreal injection		
Anti-VEGF		186 (90.7)
Bevacizumab		127 (62.0)
Ranibizumab		94 (45.9)
Triamcinolone		9 (4.4)
Laser		72 (35.1)
PRP		27 (13.2)
Focal		54 (26.3)

Abbreviations: *DEX implant* dexamethasone intravitreal implant, *VEGF* vascular endothelial growth factor, *PRP* panretinal photocoagulation.

The total number of intravitreal injections (DEX implant, anti-VEGF, or triamcinolone) received during the study period was significantly lower for patients in the monotherapy group (who received only DEX implant injections) than in the combination therapy group. Patients in the monotherapy group received 3.1 ± 1.5 (mean ± SD) intravitreal injections compared with 6.1 ± 2.8 intravitreal injections in the combination therapy group (*P* < 0.001). When the 9 patients in the combination therapy group who received intravitreal triamcinolone were excluded from the analysis, the mean total number of intravitreal injections (DEX implant plus anti-VEGF) received by patients in the combination therapy group was still 6.0 ± 2.8 (*P* < 0.001 vs. monotherapy).

### Efficacy analysis

**Figure 1:**
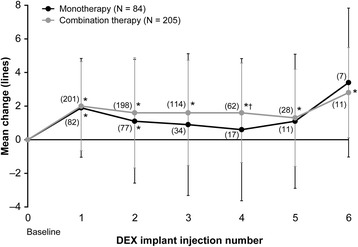
**Mean change in best-corrected visual acuity from baseline after each dexamethasone intravitreal implant (DEX implant).** Numbers in parentheses indicate number (n) of patients. Error bars, standard deviation. *P ≤ 0.026 vs. baseline, †*P* = 0.037 vs. monotherapy.

Visual acuity and CRT generally improved similarly in both groups. After the first through sixth DEX implant injections, the mean change in BCVA from baseline ranged from +0.6 to +3.4 lines in the monotherapy group and from +1.3 to + 2.8 lines in the combination therapy group (Figure 1). The between-group difference in change from baseline BCVA was statistically significant, favoring combination therapy, only after the fourth implant (monotherapy: 0.6 lines, n = 17; combination therapy: 1.6 lines, n = 62; *P* = 0.037).

**Figure 2:**
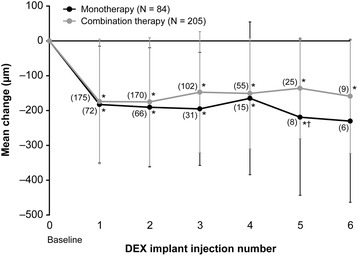
**Mean change in central retinal thickness from baseline after each dexamethasone intravitreal implant (DEX implant).** Numbers in parentheses indicate number (n) of patients. Error bars, standard deviation. *P ≤ 0.028 vs. baseline, †*P* = 0.041 vs. combination therapy.

The mean change in CRT from baseline ranged from −165 to −230 μm in the monotherapy group and from −136 to −175 μm in the combination therapy group after the first through sixth DEX implant injections (Figure 2). Improvement in CRT from baseline was statistically significant after the first through fifth implants in the monotherapy group (*P* ≤ 0.028) and after the first through sixth implants in the combination therapy group (*P* ≤ 0.020). The between-group difference in change from baseline CRT was statistically significant only after the fifth implant, favoring monotherapy (monotherapy: −219 μm; combination therapy: −136 μm; *P* = 0.041). The percentage of patients achieving CRT ≤250 μm was similar and not significantly different between the monotherapy (63.1%) and combination therapy (65.9%) groups.

**Figure 3:**
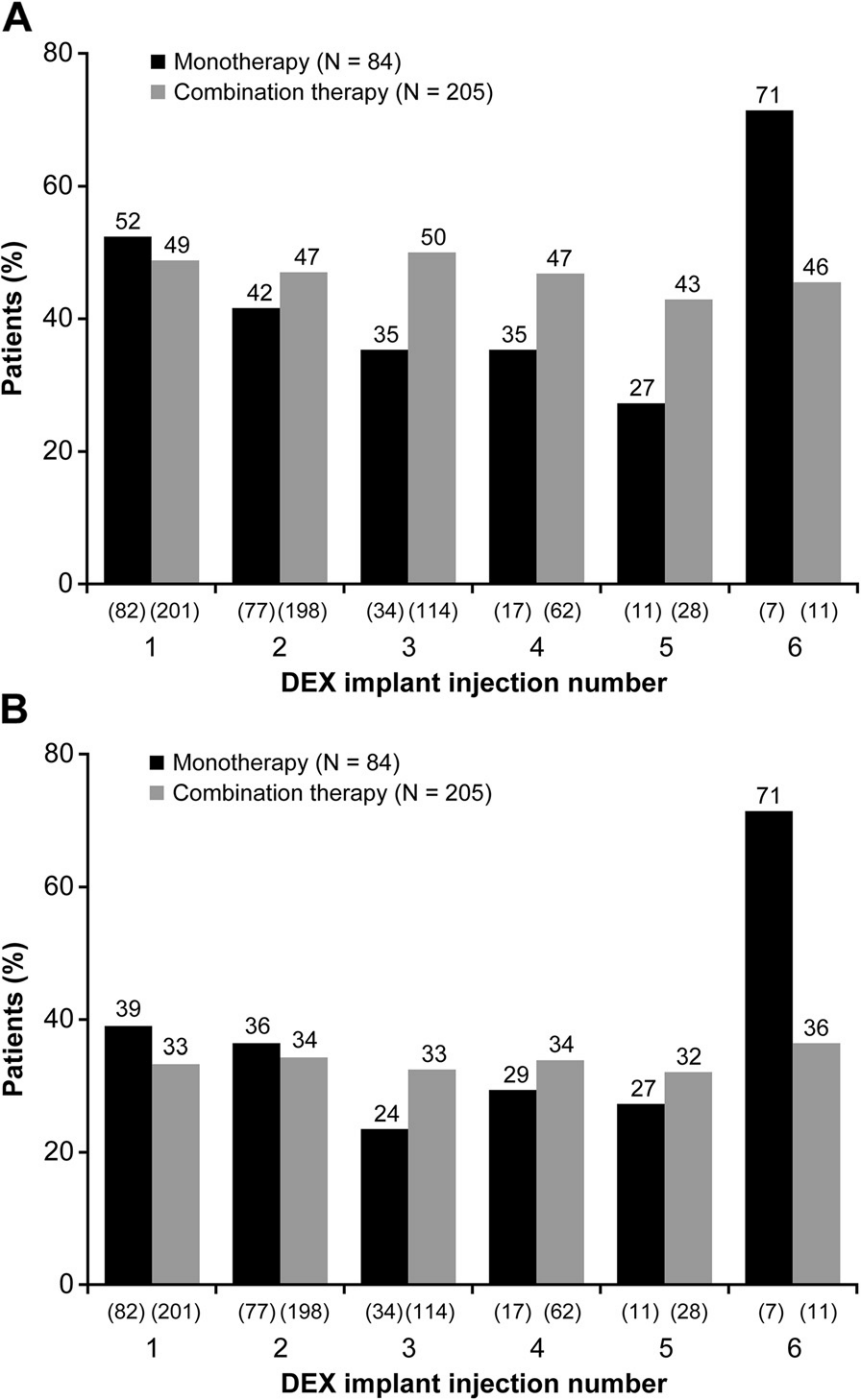
**Improvement in best-corrected visual acuity of at least 2 or 3 lines.** The percentage of patients who gained at least **(A)** 2 lines or **(B)** 3 lines in best-corrected visual acuity from baseline after each dexamethasone intravitreal implant (DEX implant) injection is shown. Numbers in parentheses indicate number (n) of patients.

**Figure 4:**
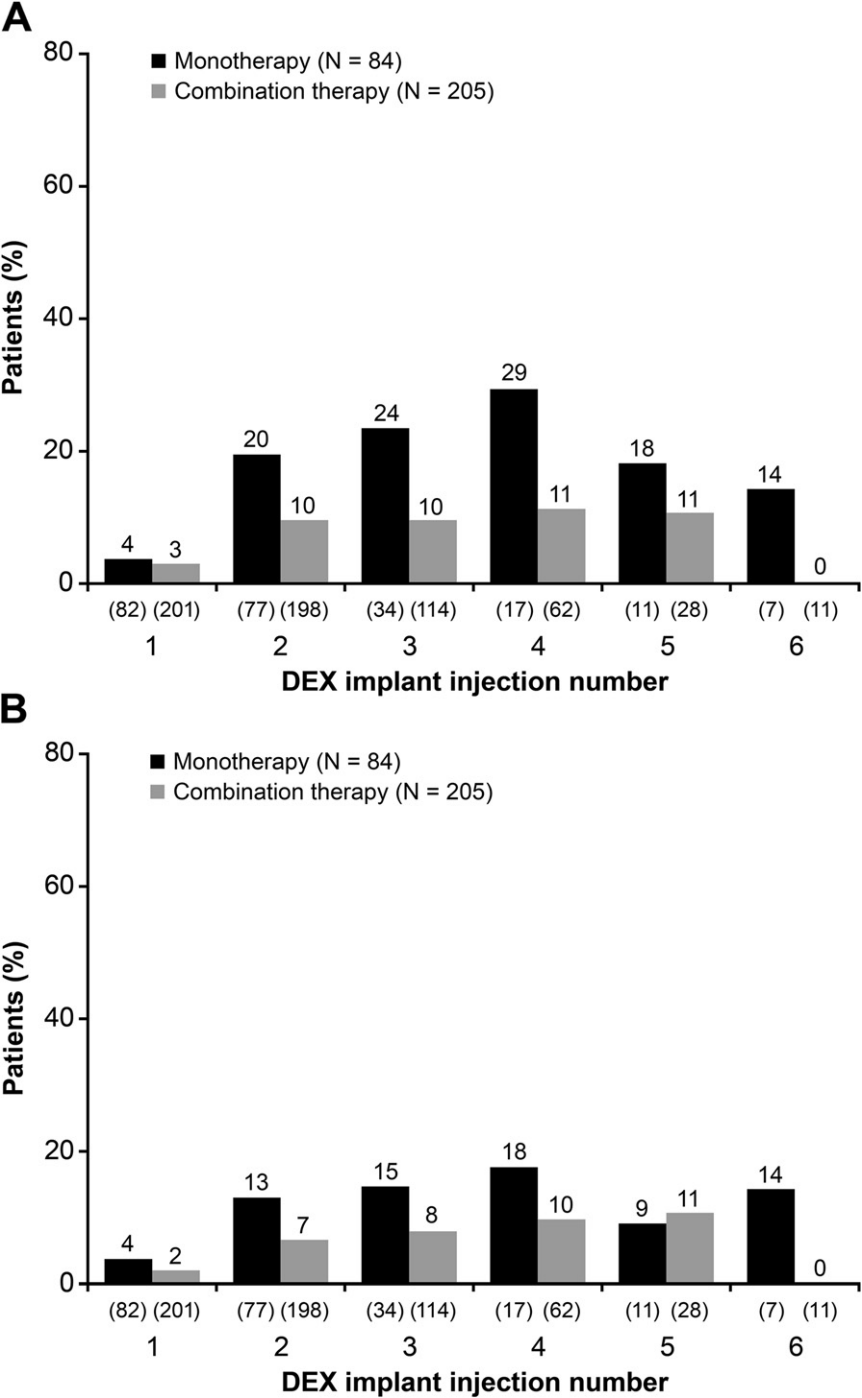
**Worsening in best-corrected visual acuity of at least 2 or 3 lines.** The percentage of patients who had at least **(A)** 2 lines or **(B)** 3 lines loss in best-corrected visual acuity from baseline after each dexamethasone intravitreal implant (DEX implant) injection is shown. Numbers in parentheses indicate number (n) of patients.

There were no significant differences between groups in the percentage of patients achieving at least 2-line or 3-line improvement in BCVA from baseline after DEX implant treatment (Figure 3). In both the monotherapy and combination therapy groups, each subsequent DEX implant demonstrated similar efficacy in improving BCVA. The percentage of patients who gained at least 3 lines in BCVA from baseline ranged from 24% to 39% after the first through fifth DEX implant injections in patients treated with DEX implant alone, and from 32% to 34% in patients treated with combination therapy. After the sixth DEX implant, 71% of patients in the monotherapy group had at least 3-line improvement in BCVA from baseline, but the number of patients was small (n = 7). Overall during the study period, 61.0% of monotherapy patients and 63.7% of combination therapy patients gained at least 2 lines in BCVA, and 52.4% of monotherapy patients and 46.3% of combination therapy patients gained at least 3 lines in BCVA. These differences between groups were not statistically significant. The percentage of patients whose peak BCVA after DEX implant treatment was at least a 2-line or 3-line loss in BCVA from baseline is shown in Figure 4.

### Safety analysis

**Figure 5:**
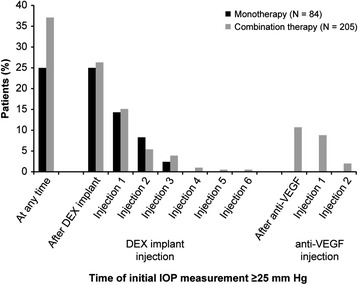
**Timing of first increase in intraocular pressure (IOP) to ≥25 mm Hg by injection number.** DEX implant, dexamethasone intravitreal implant; VEGF, vascular endothelial growth factor.

As in the total patient population [15], increase in IOP was the most common adverse event in each subgroup. For each IOP safety parameter evaluated, findings were consistently more favorable in the monotherapy group than in the combination therapy group (Table 3). An increase in IOP from baseline of at least 10 mm Hg occurred in 20 (23.8%) patients in the monotherapy group and 71 (34.6%) in the combination therapy group (*P* = 0.072), and IOP of 25 mm Hg or higher occurred in 21 (25.0%) patients in the monotherapy group and 76 (37.1%) in the combination therapy group (*P* = 0.048). Among the 76 patients in the combination therapy group who had an IOP measurement of 25 mm Hg or higher, 35 (46.1%) received ranibizumab treatment (mean of 2.4 injections) and 47 (61.8%) received bevacizumab treatment (mean of 2.4 injections) as well as DEX implant. Analysis of the timing of the initial IOP measurement ≥25 mm Hg showed that approximately one-fourth of patients in the monotherapy and combination therapy groups had IOP ≥25 mm Hg after a DEX implant injection (Figure 5). An additional 11% of patients in the combination therapy group had IOP ≥25 mm Hg after an anti-VEGF injection; IOP ≥25 mm Hg was measured in these patients at a mean of 9 days (range, 1–68 days) after their first or second anti-VEGF injection and 105 days (range, 28–164 days) after their most recent DEX implant injection. Among the 22 patients who had IOP ≥25 mm Hg after an anti-VEGF injection, 13 of them (59%) also had IOP ≥25 mm Hg after a subsequent anti-VEGF injection. The incidence of postbaseline IOP ≥25 mm Hg was 35.6% (26/73) among patients who primarily received ranibizumab and 37.7% (40/106) among patients who primarily received bevacizumab as anti-VEGF adjunctive treatment with DEX implant. Results were similar when the analysis excluded the 9 patients in the combination therapy group who received intravitreal triamcinolone during the study period. There was no significant correlation between the total number of intravitreal injections during the study period and the IOP change from baseline at the final visit for patients in the total study population or in either subgroup. Further, within each subgroup, there was no significant difference in the mean total number of intravitreal injections between patients who did and did not have IOP ≥25 mm Hg during the study period.

**Table 3:** **Intraocular pressure safety parameters**

**Parameter, n (%)**	**Monotherapy n = 84**	**Combination therapy n = 205**	***P*** **Value**
At any time during study			
Increase from baseline ≥10 mm Hg	20 (23.8)	71 (34.6)	0.072
Post-baseline IOP ≥25 mm Hg	21 (25.0)	76 (37.1)	0.048
Post-baseline IOP ≥35 mm Hg	5 (6.0)	22 (10.7)	0.205
At final visit			
Increase from baseline ≥10 mm Hg	3 (3.6)	9 (4.4)	>0.999
IOP ≥25 mm Hg	3 (3.6)	11 (5.4)	0.764
IOP ≥35 mm Hg	1 (1.2)	4 (2.0)	>0.999
Glaucoma surgery (laser/incisional)	1 (1.2)	8 (3.9)	0.455
Laser	1 (1.2)	3 (1.5)	>0.999
Incisional	0 (0.0)	5 (2.4)	0.326
IOP-lowering medication used secondary to retina treatment	20 (23.8)	64 (31.2)	0.208

Abbreviation: *IOP* intraocular pressure.

Patients with a history of IOP response to steroid appeared to be slightly more likely to have increased IOP during the study period (Table 4), but none of the parameters of increased IOP were statistically significantly different between patients with a record of history of IOP response to steroid and patients without such history. IOP-lowering medication was used secondary to retina treatment in 23.8% of patients in the monotherapy group and 31.2% of patients in the combination therapy group (*P* = 0.208).

**Table 4:** **Intraocular pressure increases during the study period in patients with or without a history of intraocular pressure response to steroid**

**History of IOP response**	**IOP increases ≥10 mm Hg, n (%)**	**Postbaseline IOP ≥25 mm Hg, n (%)**	**Postbaseline IOP ≥35 mm Hg, n (%)**
*Monotherapy group*			
Yes (n = 12)	3 (25.0)	6 (50.0)	1 (8.3)
No (n = 42)	10 (23.8)	10 (23.8)	4 (9.5)
*Combination therapy group*			
Yes (n = 33)	13 (39.4)	14 (42.4)	4 (12.1)
No (n = 126)	41 (32.5)	42 (33.3)	12 (9.5)

Abbreviation: *IOP* intraocular pressure.

One (1.2%) patient treated with DEX implant monotherapy underwent glaucoma laser surgery during the study period. In comparison, among patients treated with DEX implant plus additional RVO therapy, 3 (1.5%) underwent glaucoma laser surgery and 5 (2.4%) underwent glaucoma incisional surgery during the study period. At the final study visit, 3 (3.6%) patients in the monotherapy group and 11 (5.4%) in the combination therapy group had an IOP of 25 mm Hg or higher. There was no significant correlation between the total number of DEX implant and anti-VEGF treatments received and the IOP at the final study visit.

Cataract extractions were performed in 7.1% of patients in the monotherapy group and 19.5% of patients in the combination therapy group (*P* = 0.009). Among patients who had cataract surgery during the study period, the mean time from the first DEX implant to surgery was 257 days (range, 71–449 days) for patients treated with monotherapy and 312 days (range, 62–582 days) for patients treated with combination therapy. The difference between groups in time to cataract surgery was not statistically significant.

## Discussion

This subgroup analysis evaluated treatment efficacy, the burden of therapy, and associated morbidity in patients treated with DEX implant alone or in combination with other treatments for complications of RVO. The duration of ME prior to beginning DEX implant treatment was longer for patients who used DEX implant as monotherapy than for patients who used DEX implant in combination therapy, and the interval between DEX implant injections was shorter for patients who used DEX implant as monotherapy compared with patients who used DEX implant in combination therapy. The results showed similar gains in BCVA and reduction in CRT in both groups. The overall number of intravitreal injections and the associated costs were lower in the DEX implant monotherapy group than in the combination therapy group, and the incidence of increased IOP was the same or lower in patients treated with DEX implant monotherapy compared with patients treated with combination therapy.

A number of randomized controlled phase 3 studies (e.g., BRAVO [6] and CRUISE [8]) have demonstrated the effectiveness of intravitreal anti-VEGF injections for treatment of ME associated with RVO. It is difficult to compare results among these studies because of differences in the study populations, evidenced by differing response rates to sham treatment among studies [6,8]. The SHASTA study population was not comparable to the study populations in the anti-VEGF trials, primarily because the average duration of RVO was much longer (approximately 2 years in SHASTA versus approximately 3 months in BRAVO and CRUISE). Nonetheless, the data in SHASTA represent actual use patterns, rather than the fixed retreatment interval of 6 months used in the DEX implant phase 3 studies; therefore, interpretation of the SHASTA study results in light of those from the anti-VEGF trials may be useful.

Mean BCVA at baseline was worse among patients with RVO treated with DEX implant monotherapy in the SHASTA study (~20/125) than in BRVO patients treated with ranibizumab in the BRAVO study (~20/80) [6] or CRVO patients treated with ranibizumab in the CRUISE study (~20/100) [8], and the duration of ME at baseline was much longer in the SHASTA study. Even so, visual outcomes in SHASTA were as favorable as those in BRAVO and CRUISE, with a similar percentage of patients gaining 3 lines or more in BCVA. At month 3 (1 month after the third monthly ranibizumab injection) 50.4% of patients in BRAVO and 36.9% of patients in CRUISE had gained at least 3 lines in BCVA. In comparison, 39.9% of patients treated with DEX implant monotherapy in SHASTA gained at least 3 lines in BCVA after the first DEX implant injection, and 52.4% gained at least 3 lines in BCVA during the study period.

Combination of therapies with different mechanisms of action frequently leads to increased treatment effectiveness. In the present analysis, the proportion of patients with BRVO versus CRVO and the likelihood of having received previous treatment for RVO were similar between groups, but a significantly higher percentage of patients in the combination therapy group compared with the monotherapy group had received anti-VEGF treatment prior to their first DEX implant (75.1% vs. 60.7%, *P* = 0.014). Preferential inclusion of patients with persistent edema despite multiple anti-VEGF injections in the combination therapy group may explain the observed lack of increased treatment effectiveness of combination therapy in the present analysis. On the other hand, a significantly higher percentage of patients in the monotherapy group compared with the combination therapy group had received focal laser treatment prior to the first DEX implant (39.3% vs. 25.4%, *P* = 0.018); 26.3% of patients in the combination therapy group also received focal laser treatment during the study. Further, at the time of the first DEX implant, the CRT was numerically thicker in the monotherapy group (465 μm) than in the combination therapy group (427 μm), and the duration of ME was significantly longer in the monotherapy group. Since ME of longer duration is less amenable to treatment [10-12], this difference in duration of RVO would have been expected to result in less favorable outcomes in the monotherapy group.

Patients may benefit from using a single injection of DEX implant that lasts significantly longer than anti-VEGFs [15-17] rather than monthly injections of other treatments, as frequent office visits place a greater burden on patients and increase costs. DEX implant monotherapy was more cost-effective than combination therapy in the present analysis, as DEX implant alone provided similar effectiveness as combination therapy, with similar or lower morbidity and a lower burden of treatment and costs. If similar visual outcomes can be obtained, with less frequent injections overall, there is added value in using DEX implant as monotherapy, especially given the morbidities and adverse events now being attributed to anti-VEGFs [18,19].

The difference between groups in the incidence of increased IOP is provocative. There was no statistically significant difference between the monotherapy and combination therapy groups at baseline with respect to the percentage of patients diagnosed with glaucoma or ocular hypertension or using IOP-lowering medication. The percentage of patients with a history of IOP rise in response to steroid was also similar in the two groups. During the study period, the only RVO treatment received by patients in the monotherapy group was DEX implant, whereas 90.7% of patients in the combination therapy group received anti-VEGF and 4.4% received intravitreal triamcinolone. The mean number of DEX implants received by patients was ~3 in both groups, yet a significantly higher percentage of patients in the combination therapy group compared with the monotherapy group had IOP increase to 25 mm Hg or higher (37.1% vs. 25.0%, *P* = 0.048), and patients in the combination therapy group were also more likely than patients in the monotherapy group to have IOP increase by at least 10 mm Hg (34.6% vs. 23.8%, *P* = 0.072). The difference between groups in the occurrence of IOP increases was evident even when patients who received intravitreal triamcinolone during the study period were excluded from the analysis. There was also a trend for greater use of IOP-lowering medication secondary to the retina treatment in the combination therapy group. These findings are consistent with previous reports of the occurrence of a sustained increase in IOP in up to 11% of eyes after multiple intravitreal anti-VEGF injections [20-25]. The median number of anti-VEGF injections before increases in IOP were observed reportedly ranged from 5 to 17 [21,22,25]. There is conflicting evidence as to whether more frequent anti-VEGF injections increase the risk of IOP rises [21,22]. However, sustained increases in IOP during intravitreal anti-VEGF treatment seem most likely to occur in patients with preexisting glaucoma or ocular hypertension [23], and overall, patients who receive a greater number of anti-VEGF injections at shorter injection intervals appear to be at greater risk of a sustained increase in IOP [23].

In the present subgroup analysis of the SHASTA study, the increased occurrence of IOP increases in the combination therapy group compared with the monotherapy group resulted primarily from IOP increases occurring after anti-VEGF injections. The results of the analysis of the timing of IOP increases are consistent with a hypothesis that a greater overall number and frequency of intravitreal injections increases the risk of increased IOP. However, as the mean total number of intravitreal injections during the study period was similar in patients who had increased IOP and those who did not, use of anti-VEGF treatment may better explain the increased occurrence of IOP rises in the combination therapy group compared with the monotherapy group.

A history of IOP response to steroid did not significantly increase the likelihood of IOP increases in either the monotherapy group or the combination therapy group. Accordingly, most patients with a history of IOP response to steroid had no recorded increase in IOP ≥10 mm Hg or IOP measurement ≥25 mm Hg after DEX implant treatment. Although steroid-induced increases in IOP are receptor mediated, different intravitreal steroid treatments do not have equivalent effects on IOP. Dexamethasone is a potent steroid, yet DEX implant is associated with a lower incidence of increases in IOP compared with the intravitreal fluocinolone implant or intravitreal triamcinolone [26]. The cellular effects and pharmacokinetics of the different intravitreal steroid treatments account for their differing safety profiles. Dexamethasone, fluocinolone, and triamcinolone activate different patterns of gene expression in human trabecular meshwork cell lines [27]. Further, less lipophilic steroids (e.g., dexamethasone compared with triamcinolone and fluocinolone) partition less to the trabecular meshwork and lens, and are therefore associated with a lower incidence of IOP increases and cataract progression [28]. The sustained release of dexamethasone from DEX implant also contributes to its favorable safety profile. A sustained-release implant and a bolus injection of steroid would not be expected to have the same effect on IOP: there is less risk with repeated injections of DEX implant.

This study has the limitations common to retrospective chart review analyses. In particular, selection bias may have influenced the comparison of results between the monotherapy and combination therapy groups. Eyes treated with monotherapy may represent eyes that had a good initial response to DEX implant and may be different in nature to eyes in the combination therapy group. Further, the patient selection requirement of at least 2 DEX implants might have affected the results in either a negative or a positive manner, as patients who were not retreated, either because they had resolution of ME after a single DEX implant or single DEX implant combination treatment, or because they had poor response to a single DEX implant, were excluded. Also, retrospective data prior to the first DEX implant treatment were not collected. Finally, in the analyses of BCVA and CRT after each subsequent implant, the number of patients decreased substantially after the third implant. The effects of later implants were consistent with those of earlier implants, however.

## Conclusions

This study demonstrated that treatment with 2 or more DEX implants is safe and effective in the treatment of RVO-associated ME when used alone, as well as when used in combination with other RVO treatments. Use of DEX implant alone at 4 to 5 month intervals is effective in many patients and less burdensome to patients than combining DEX implant with anti-VEGF treatment. In this study, DEX implant treatment was safe and effective even in patients with a history of IOP response to steroid. Increases in IOP that occurred were usually controlled with topical medication. When combining treatments for RVO-associated ME, physicians should consider the possibility of IOP increases related to the total number and frequency of intravitreal injections.
